# Exercise testing and postoperative complications after minimally invasive lung resection: A cohort study

**DOI:** 10.3389/fphys.2022.951460

**Published:** 2022-09-23

**Authors:** Gabriel Chouinard, Pascalin Roy, Marie-Christine Blais, Alexandre Lippens, Éliane Pelletier, Emma Roy, Mathieu Marcoux, Paula A. Ugalde, Justine Rheault, Marc-Antoine Pigeon, Frédéric Nicodème, Yves Lacasse, François Maltais

**Affiliations:** ^1^ Institut universitaire de cardiologie et de pneumologie de Québec, Université Laval, Québec, QC, Canada; ^2^ Centre hospitalier universitaire de Québec, Université Laval, Québec, QC, Canada

**Keywords:** post-operative outcomes, cardiopulmonary exercise, peak oxygen consumption (peak VO2), thoracoscopy (VATS), lung cancer, lung resection

## Abstract

**Background:** Peak oxygen uptake ( $\dot{V}O_{2}$ ) during cardiospulmonary exercise testing (CPET) is used to stratify postoperative risk following lung cancer resection but peak $\dot{V}O_{2}$ thresholds to predict post-operative mortality and morbidity were derived mostly from patients who underwent thoracotomy.

**Objectives:** We evaluated whether peak $\dot{V}O_{2}$ or other CPET-derived variables predict post-operative mortality and cardiopulmonary morbidity after minimally invasive video-assisted thoracoscopic surgery (VATS) for lung cancer resection.

**Methods:** A retrospective analysis of patients who underwent VATS lung resection between 2002 and 2019 and in whom CPET was performed. Logistic regression models were used to determine predictors of postoperative outcomes until 30 days after surgery. The ability of peak $\dot{V}O_{2}$ to discriminate between patients with and without post-operative complications was evaluated using Receiver operating characteristic (ROC) analysis.

**Results:** Among the 593 patients, postoperative cardiopulmonary complications occurred in 92 (15.5%) individuals, including three deaths. Mean peak $\dot{V}O_{2}$ was 18.8 ml⋅kg^−1^⋅min^−1^, ranging from 7.0 to 36.4 ml⋅kg^−1^⋅min^−1^. Best predictors of postoperative morbidity and mortality were peripheral arterial disease, bilobectomy or pneumonectomy (versus sublobar resection), preoperative FEV_1_, peak $\dot{V}O_{2}$ , and peak $\dot{V}E/\dot{V}CO_{2}$ . The proportion of patients with peak $\dot{V}O_{2}$ of < 15 ml⋅kg^−1^⋅min^−1^, 15 to < 20 ml⋅kg^−1^⋅min^−1^ and ≥ 20 ml⋅kg^−1^⋅min^−1^ experiencing at least one postoperative complication was 23.8, 16.3 and 10.4%, respectively. The area under the ROC curve for peak $\dot{V}O_{2}$ was 0.63 (95% CI: 0.57–0.69).

**Conclusion:** Although lower peak $\dot{V}O_{2}$ was a predictor of postoperative complications following VATS lung cancer resection, its ability to discriminate patients with or without complications was limited.

## Introduction

Lung cancer is currently the leading cause of death by cancer (Cancer IAfRo, 2019). Although surgery still offers the best chance to cure early stage non-small-cell lung cancer (Howington et al., 2013), pre-operative assessment and stratification of lung resection candidates is a crucial step to minimize surgical risks as this population is frequently older and comorbid (Sekine et al., 2002; Kravchenko et al., 2015).

Cardiopulmonary exercise testing (CPET) has played a central role in the evaluation of fitness of lung resection candidates. This is particularly true in patients with impaired lung function in whom various peak oxygen uptake (peak $\dot{V}O_{2}$ ) thresholds have been shown to predict increased mortality and morbidity risk following lung resection (Bolliger et al., 1995; Brutsche et al., 2000; Win et al., 2005; Loewen et al., 2007; Brunelli et al., 2009a; Fang et al., 2014; Rodrigues et al., 2016). Peak $\dot{V}O_{2}$ thresholds have thus been incorporated into clinical algorithms that stratify patients into low, moderate, and high risk of poor post-operative outcomes (Brunelli et al., 2013).

An important issue with this practice is that peak $\dot{V}O_{2}$ thresholds to predict post-operative mortality and morbidity were derived from patients who underwent thoracotomy. Video-assisted thoracic surgery (VATS) has now become widely available and the recommended surgical approach for the majority of lung resections as it is associated with less complications compared to thoracotomy (Paul et al., 2013; Laursen et al., 2016). Whether peak $\dot{V}O_{2}$ or any other CPET variables would still predict post-operative morbidity in this era where minimally invasive surgical procedures and improved anesthetic techniques have been implemented is uncertain.

The aim of this study was to assess if clinical and physiological variables, including peak $\dot{V}O_{2}$ , predict post-operative mortality and cardiopulmonary morbidity in patients undergoing VATS lung cancer resection. Considering the minimally invasive nature of the surgical approach in the context of improved peri-operative care, our hypothesis was that CPET variables would not be highly predictive of post-operative outcomes in this specific population.

## Methods

### Subjects and study design

This is a retrospective analysis of a prospectively collected research database of all patients with a resectable lung cancer who performed CPET as part of their preoperative medical evaluation at the *Institut universitaire de cardiologie et de pneumologie de Québec* (IUCPQ), Québec, Canada. Patients who underwent VATS lung cancer surgery from 1 January 2002 to 31 December 2019 were included on a first occurrence of a non-small-cell lung cancer without prior thoracic surgery or radiotherapy and if they had pulmonary function testing and CPET data available. Surgeries were performed by thoracic surgeons at the IUCPQ where VATS progressively became the preferred approach. In 2005, VATS represented 25% of all lung cancer resections compared to 88% in 2019. CPET was also requested by the surgeons based on their clinical judgment that further testing was necessary to complete surgical risk assessment. When lung resection was considered as a potential therapeutic option, the following situations would generally trigger a CPET: forced expiratory volume in 1 s (FEV_1_) < 80% predicted, age > 65 years, the presence of one or more comorbidities, and the possibility of lung resection more extensive than a lobectomy. All study participants provided informed consent and gave written approval for the use of their clinical data in subsequent research publication (CER 21184).

The primary outcome of the study was cardiopulmonary complications occurring during the 30-day period following the surgery. Baseline characteristics, pulmonary function tests, CPET, and 30-days post-operative cardiopulmonary complications and mortality were retrieved from the research database and review of the medical chart, when necessary. Age, sex, body mass index (BMI), smoking history, and extent of resection (pneumonectomy, bilobectomy, lobectomy, sublobar resection) were noted. The following comorbid conditions were documented: coronary artery disease, peripheral artery disease, hypertension, diabetes, chronic renal failure, and chronic obstructive pulmonary disease (COPD). COPD diagnosis was based on the presence of symptoms related to COPD, spirometry showing a post-bronchodilator FEV_1_ to forced vital capacity (FVC) ratio < 0.70, and a smoking history of at least 10 pack-years (Vogelmeier et al., 2017). The Thoracic Revised Cardiac Risk Index (ThRCRI) was calculated for each patient (Brunelli et al., 2010). This index provides a summary score that stratifies patients into four classes, from A to D, with a progressively higher risk of post-operative cardiovascular complications.

Pulmonary function tests, including spirometry, lung volumes, and carbon monoxide diffusion capacity were performed according to previous guidelines and related to predicted normal values (Macintyre et al., 2005; Miller et al., 2005; Wanger et al., 2005). CPET results were reviewed and then adjudicated by a second author (GC, AL, and PR). Patients with CPET judged to be non-maximal according to the American College of Sports Medicine statement on CPET (Swain et al., 2000) were excluded. Briefly, when CPET was stopped before the predicted peak $\dot{V}O_{2}$ was achieved, the test was considered non-maximal when maximal heart rate was not within 10 beats of the estimated maximum heart rate, minute ventilation ( $\dot{V}E$ )/maximum voluntary ventilation was < 85%, respiratory exchange ratio was < 1.10, and perceived exertion did not reach 7 on a 0–10 Borg scale.

Grade II or more post-operative cardiopulmonary complications on the Clavien-Dindo (Dindo et al., 2004) classification scheme (those requiring specific interventions beyond antipyretics, antiemetic, and analgesic) were recorded and definitions were based on the Society of Thoracic Surgeons and European Society of Thoracic Surgeons joint statement (Dindo et al., 2004; Fernandez et al., 2015). The following cardiopulmonary complications were recorded: need for mechanical ventilation, pneumonia, acute respiratory distress syndrome (ARDS), atelectasis, acute coronary syndrome, arrythmia, heart failure, venous thromboembolism, stroke, and acute renal failure.

### Cardiopulmonary exercise testing

CPET consisted in an incremental cycling exercise performed on a cycle ergometer, until exhaustion and were performed at the clinical exercise laboratory of our institution. After 1 minute of resting on the cycle ergometer, work rate was increased in a ramp protocol using of 10- to 20-W per minute increments, with the objective of having an exercise duration of 8–10 min based on predicted peak work rate. $\dot{V}O_{2}$ , carbon dioxide excretion ( $\dot{V}CO_{2}$ ) and $\dot{V}E$ were monitored by a commercial breath-by-breath exercise circuit. Heart rate (HR), and O_2_ pulse saturation (SpO_2_) were continuously monitored through a 12-lead electrocardiogram and a pulse oximeter, respectively. Peak workrate and peak $\dot{V}O_{2}$ were expressed in abolute units and in % predicted values according to the following formulas developed by Jones and Carol (1988): Peak workrate = (25.26*height—9.08*age—2,759)*0.16344; Peak $\dot{V}O_{2}$ = 0.0541*height—0.025*age - 5.66; in women: Peak workrate = (12.66*height—8.27*age—940)*0.16344; Peak $\dot{V}O_{2}$ = 0.0301*height—0.017*age—2.56, where workrate is expressed in watts, height in cm, and age in years. Predicted peak heart rate was calculated as 210–0.65 X age in years and maximal voluntary ventilation was calculated as FEV_1_ X 35 (Jones and Carol, 1988).

### Statistical analysis

Results are reported as mean ± SD for continuous variables and number or % for nominal variables. Between-group comparisons were performed using the Student’s t-test for continuous variables and Chi-Square or Fisher’s exact test for nominal variables. We conducted univariable logistic regression analyses using potential predictors of post-surgical outcomes (age, sex, comorbid conditions, ThRCRI class C and D, extent of lung resection, FEV_1_% predicted, FVC % predicted, FEV_1_/FVC, total lung capacity % predicted, residual volume % predicted, carbon monoxide diffusion capacity [DLCO] % predicted, peak values for $\dot{V}O_{2}$ expressed in ml⋅kg^−1^⋅min^−1^ and in % predicted, $\dot{V}E$ , $\dot{V}E/\dot{V}CO_{2}$ , heart rate, SpO_2_) and a composite of cardiopulmonary morbidity and mortality as independent variables. Dependant variables that were associated with post-operative outcome with a *p* < 0.20 in the univariable analysis were then incorporated in a multivariable logistic regression model. Receiver operating characteristic (ROC) curve analysis was used to determine the ability of peak $\dot{V}O_{2}$ expressed in ml⋅kg^−1^⋅min^−1^ to discriminate between patients who did nor did not experience post-operative complications. This was done by measuring the area under the ROC curve and the value at which the Youden’s index (true positive rate + true negative rate -1) is maximal. An area under the ROC curve of 0.5 indicates no discrimination while values ranging from 0.7 to 0.8, 0.8 to 0.9, and > 0.9 are considered acceptable, excellent, and outstanding, respectively (Mandrekar, 2010). To determine the ability of peak $\dot{V}O_{2}$ expressed in ml⋅kg^−1^⋅min^−1^ to predict specific types of post-operative complications, multivariable logistic models and ROC curve analyses were also conducted for: 1) pulmonary complications only, 2) cardiac complications only, and 3) grade III or more post-operative cardiopulmonary complications on the Clavien-Dindo classification scheme (Dindo et al., 2004) (those requiring surgical, endoscopic, or radiological interventions) only.

## Results

Baseline characteristics of the study participants are summarized in Table 1. From 1 January 2002 to 31 December 2019, 2,228 patients underwent lung cancer resection by VATS at our institution. The study population included the 593 patients who performed a CPET pre-operatively, representing 27% of the entire VATS population. The study population included 264 men (44.5%), mean age was 66.8 years and BMI averaged 26.9 kg/m^2^. COPD, hypertension, coronary artery disease, and diabetes were the most frequent comorbid conditions. Mean peak work rate was 91.4% of predicted and mean peak $\dot{V}O_{2}$ was 18.8 ml⋅kg^−1^⋅min^−1^ with a range of 7.0–36.4 ml⋅kg^−1^⋅min^−1^, including four patients with a peak $\dot{V}O_{2}$ < 10 ml⋅kg^−1^⋅min^−1^.

**TABLE 1:** Demographic, surgical, spirometric and cardiopulmonary exercise test data (*n* = 593).

	Population	Without Cardiopulmonary complication	With Cardiopulmonary complication	p Value
	*n* = 593	*n* = 501	*n* = 92	
**Demography**				
Male, sex*, n* (%)	264 (44.5)	222 (44.3)	42 (45.7)	0.820
Age, years	66.8 ± 7.4	66.5 ± 7.6	68.5 ± 6.2	0.007
Body mass index, kg/m^2^	26.9 ± 5.6	27.2 ± 5.6	26.3 ± 5.6	0.095
CAD, *n* (%)	114 (19.3)	93 (18.6)	21 (22.8)	0.388
PAD*, n* (%)	98 (16.5)	76 (15.2)	22 (23.9)	0.047
Hypertension*, n* (%)	318 (53.6)	259 (51.7)	59 (64.1)	0.031
Diabetes, *n* (%)	110 (18.6)	88 (17.6)	22 (23.9)	0.188
COPD*, n* (%)	330 (55.7)	273 (54.5)	57 (62.0)	0.210
eGFR*,* ml/min/1.73 m (Sekine et al., 2002)	80.3 ± 16.8	80.2 ± 16.6	80.6 ± 17.9	0.728
ThRCRI ≥ 2, *n* (%)	38 (6.4)	26 (5.2)	12 (13.0)	0.009
**Surgery**				
Pneumonectomy, *n* (%)	20 (3.4)	11 (2.2)	9 (9.8)	0.002
Bilobectomy, *n* (%)	31 (5.2)	23 (4.6)	8 (8.7)	
Lobectomy, *n* (%)	440 (74.2)	378 (75.5)	62 (67.4)	
Sublobar resection, *n* (%)	102 (17.2)	89 (17.8)	13 (14.1)	
**Pulmonary function tests**				
FEV_1_, L	2.0 ± 0.6	2.0 ± 0.6	1.9 ± 0.6	0.007
FEV_1_, % predicted	80.0 ± 19.0	80.9 ± 19.2	74.7 ± 16.7	0.002
FVC, L	3.1 ± 0.9	3.1 ± 0.9	2.9 ± 0.9	0.054
FVC, % predicted	94.6 ± 16.5	95.4 ± 16.8	90.3 ± 14.0	0.002
FEV_1_/FVC, %	65.5 ± 11.3	65.8 ± 11.3	63.8 ± 11.3	0.111
D_L_CO, % predicted	79.2 ± 23.3	80.0 ± 23.4	74.8 ± 22.0	0.049
RV, % predicted	140.3 ± 40.0	140.2 ± 40.6	140.6 ± 36.9	0.938
TLC, % predicted	108.6 ± 15.9	109.2 ± 16.1	105.9 ± 14.9	0.070
**Cardiopulmonary exercise test**				
Peak workrate, watts	100.7 ± 33.5	101.9 ± 33.8	94.3 ± 30.8	0.034
Peak workrate, % predicted	91.4 ± 21.6	92.2 ± 22.0	87.1 ± 18.8	0.021
Peak $\dot{V}O_{2}$ , ml⋅kg^−1^⋅min^−1^	18.8 ± 4.4	19.1 ± 4.5	17.3 ± 3.9	<0.001
Peak $\dot{V}O_{2}$ , % predicted	100.6 ± 28.3	101.7 ± 28.6	94.5 ± 26.0	0.017
Peak $\dot{V}E$ , L	59.3 ± 17.5	59.9 ± 17.8	56.1 ± 15.2	0.032
Peak $\dot{V}E$ /MVV	90.9 ± 24.0	90.4 ± 24.4	93.7 ± 21.6	0.143
Peak $\dot{V}E$ / $\dot{V}CO_{2}$	36.7 ± 7.2	36.3 ± 7.1	38.8 ± 7.5	0.003
SpO_2_ at rest	97.0 ± 1.9	97.0 ± 1.9	96.8 ± 2.1	0.427
SpO_2_ at peak $\dot{V}O_{2}$	95.8 ± 3.0	95.8 ± 3.1	95.7 ± 2.6	0.465
Peak heart rate, bpm	138.4 ± 19.6	139.1 ± 19.7	134.2 ± 18.4	0.022
Heart rate reserve, bpm	28.2 ± 18.6	27.7 ± 18.6	31.3 ± 18.8	0.096
Peak heart rate, % max predicted	83.0 ± 11.2	83.4 ± 11.2	81.2 ± 11.2	0.086

Definitions of abbreviations: CAD = coronary arterial disease; PAD = peripheral arterial disease; COPD = chronic obstructive pulmonary disease (based on Global Initiative for Chronic Obstructive lung disease - GOLD); eGFR = estimated glomerular filtration rate; ThRCRI = Thoracic Revised Cardiac Risk Index; from 0 to 5.5, based on history of ischemic heart disease, history of cerebrovascular disease, serum creatinine above 176 μmol/L (2 mg/dl), and undergoing a pneumonectomy. FEV_1_ = forced expiratory volume in 1 s; FVC = forced vital capacity; D_L_CO = carbon monoxide diffusion capacity; RV = residual volume; TLC = total lung capacity; Peak $\dot{V}O_{2}$ = oxygen uptake at peak exercise; Peak $\dot{V}E$ = ventilation at peak exercise; MVV = maximum voluntary ventilation; $\dot{V}CO_{2}$ = carbon dioxide excretion at peak exercise; SpO_2_ = O_2_ pulse saturation.

The overall distribution of postoperative cardiopulmonary complications according to the extent of lung resection is summarized in Table 2. Overall, 92 (15.5%) patients experienced one or more of the 130 cardiopulmonary complications within 30 days of the operation, including three deaths (0.5%), two from acute respiratory distress syndrome complicating a bronchopleural fistula and one from an acute exacerbation of pulmonary fibrosis. The most frequent complications were pneumonia (5.9%) and arrhythmias (6.8%). A larger proportion of patients who underwent bilobectomy and pneumonectomy experienced post-operative complications compared to those with less extensive resection.

**TABLE 2:** Number of post-operative cardiopulmonary complications within 30 days of surgery according to the extent of lung resection (*n* = 593).

Complications	All surgery	Bilobectomy or pneumonectomy	Lobectomy	Sublobar resection	*p*
	*n* = 593	*n* = 51	*n* = 440	*n* = 102	
**Pulmonary complications**					
Mechanical ventilation	5 (0.8)	2 (3.9)	2 (0.5)	1 (1.0)	0.041
Pneumonia	35 (5.9)	5 (9.8)	24 (5.5)	6 (5.9)	0.437
ARDS	3 (0.5)	2 (3.9)	1 (0.2)	0 (0)	0.033
Atelectasis	6 (1.0)	1 (2.0)	5 (1.1)	0 (0)	0.331
Total pulmonary complications	49 (8.3)	10 (19.6)	32 (7.3)	7 (6.9)	0.154
**Cardiac complications**					
Acute coronary syndrome	2 (0.3)	2 (3.9)	0 (0)	0 (0)	0.007
Arrhythmia	40 (6.7)	7 (13.7)	29 (6.6)	4 (3.9)	0.090
Acute heart failure	13 (2.2)	3 (5.9)	7 (1.6)	3 (2.9)	0.070
Venous thromboembolism	4 (0.7)	0 (0)	3 (0.7)	1 (1.0)	0.698
Other	19 (3.2)	4 (7.8)	11 (2.5)	4 (3.9)	0.103
Total cardiac complications	78 (13.2)	16 (31.4)	50 (11.4)	12 (11.8)	0.026
**Death**	3 (0.5)	2 (3.9)	1 (0.2)	0 (0)	0.033
**Total**	130 (21.9)	28 (54.9)	83 (18.9)	19 (18.6)	0.003

Values are n (%). A patient may have experienced more than one cardiopulmonary complication. Abbreviation: ARDS: acute respiratory distress syndrome.

Comparisons between patients with at least one post-operative cardiopulmonary complications and those who experienced an uneventful surgery are provided in Table 1. Age, peripheral artery disease, hypertension, ThRCRI ≥ 2 (class C or D), and bilobectomy or pneumonectomy were associated with complications. FEV_1_, FVC and DLCO were lower in patients with complications. Peak $\dot{V}O_{2}$ was 101.7% predicted in patients with an uneventful post-operative period compared to 94.5% in those with complications. This translated in a 1.8 ml⋅kg^−1^⋅min^−1^ difference in mean absolute peak $\dot{V}O_{2}$ (19.1 ± 4.5 versus 17.3 ± 3.9 ml⋅kg^−1^⋅min^−1^). Consistent with these results, peak work capacity was greater in patients whose surgery was uneventful, averaging 92.2% predicted versus 87.1%. Lower peak $\dot{V}E$ and peak heart rate and higher peak $\dot{V}E/\dot{V}CO_{2}$ were finally noted in patients with complications.

As reported in Table 3, the best predictors of post-operative mortality and morbidity in the multivariable analysis were peripheral artery disease (odds ratio [OR] = 1.90, 95% CI: 1.08–3.37), bilobectomy (OR = 3.44, 95%CI: 1.20 to 9.85 versus sublobar resection), pneumonectomy (OR = 10.32, 95%CI 3.29 to 32.35 versus sublobar resection), FEV_1_% predicted (OR = 0.98 per unit, 95%CI: 0.97–1.0), peak $\dot{V}O_{2}$ , ml⋅kg^−1^⋅min^−1^ (OR = 0.92 per unit, 95%CI: 0.86–0.98), and peak $\dot{V}E/\dot{V}CO_{2}$ (OR = 1.04 per unit, 95%CI: 1.01–1.07).

**TABLE 3:** Best predictors of post-operative complications based on the multivariable regression model.

Parameters	OR	95% CI
Peripheral artery disease	1.90	1.08 to 3.37
Bilobectomy (vs. sublobar)	3.44	1.20 to 9.85
Pneumonectomy (vs. sublobar)	10.32	3.29 to 32.35
Preoperative FEV_1_ (% predicted)	0.98	0.97 to 1.00
Peak $\dot{V}O_{2}$ (ml⋅kg^−1^⋅min^−1^)	0.92	0.86 to 0.98
Peak $\dot{V}E$ / $\dot{V}CO_{2}$	1.04	1.01 to 1.07

Definitions of abbreviations: OR = odds ratio; CI = confidence interval; FEV_1_ = maximal forced expiratory volume in 1 s; Peak $\dot{V}O_{2}$ = oxygen uptake at peak exercise; Peak $\dot{V}E$ = ventilation at peak exercise; $\dot{V}CO_{2}$ = carbon dioxide excretion at peak exercise.

To further explore possible relationships between peak $\dot{V}O_{2}$ and adverse post-operative outcomes, the proportion of patients experiencing at least one cardiovascular complication was documented according to categories of baseline peak $\dot{V}O_{2}$ ml⋅kg^−1^⋅min^−1^ (table 4). Only four patients (0.7%) had a peak $\dot{V}O_{2}$ < 10 ml⋅kg^−1^⋅min^−1^. Peak $\dot{V}O_{2}$ between 10 and < 15 ml⋅kg^−1^⋅min^−1^, 15 to < 20 ml⋅kg^−1^⋅min^−1^, and ≥ 20 ml⋅kg^−1^⋅min^−1^ was found in 101 (17%), 276 (47%), and 212 (36%) individuals, respectively. The proportion of patients experiencing complications in these peak $\dot{V}O_{2}$ categories was 24.8, 16.3, and 10.4%, respectively (*p* = 0.01 between groups).

**FIGURE 1:**
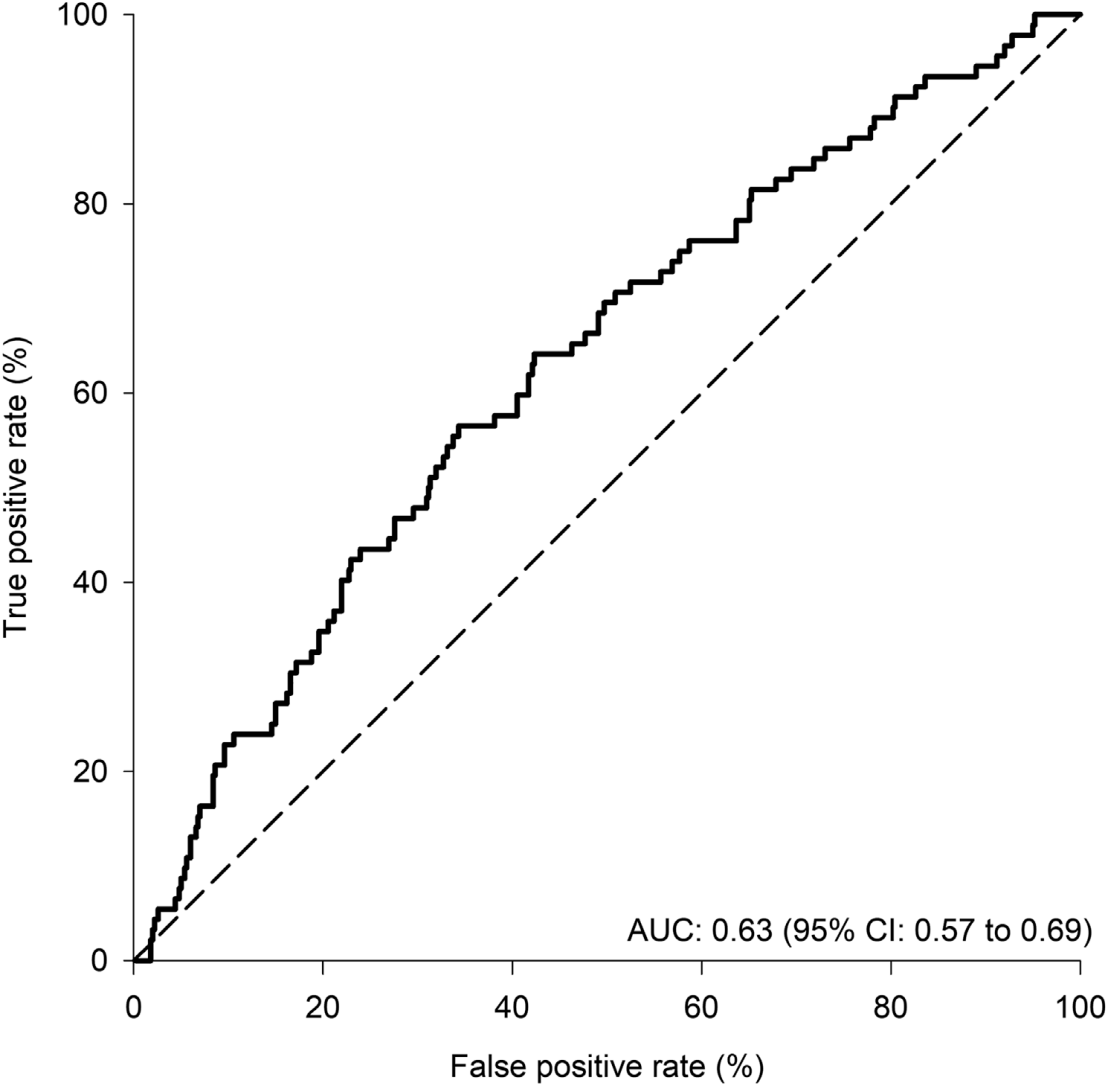
Receiver operating characteristic (ROC) curve for peak $\dot{V}O_{2}$ expressed in ml⋅kg^−1^⋅min^−1^. The dash line indicates a situation of a test that would have no discriminatory value for the occurrence of post-operative complications, with an area under the curve (AUC) of 0.5.

The ROC curve for peak $\dot{V}O_{2}$ ml⋅kg^−1^⋅min^−1^ to discriminate for the occurrence of cardiopulmonary post-operative complications in provided in Figure 1. The area under the ROC curve for peak $\dot{V}O_{2}$ was 0.63 (95% CI: 0.57–0.69) and the maximum value of the Youden’s index was 0.22 at a peak $\dot{V}O_{2}$ of 17 ml⋅kg^−1^⋅min^−1^. At this peak $\dot{V}O_{2}$ , the true positive rate i.e. the sensitivity (the proportion of patients who experienced a post-operative complication who had a peak $\dot{V}O_{2}$ < 17 ml⋅kg^−1^⋅min^−1^) was 56.5% and the false positive rate i.e., 100 – specificity % (the proportion of patients who did not experience a post-operative complication who had a peak $\dot{V}O_{2}$ < 17 ml⋅kg^−1^⋅min^−1^) was 34.3%.

The ability of peak $\dot{V}O_{2}$ ml⋅kg^−1^⋅min^−1^ to predict post-operative complications was not improved by restricting the analysis to specific complications and the results were consistent across various types of complications. The ORs of experiencing only pulmonary, only cardiac, or only grade III or more complications varied from 0.90 to 0.95 per unit of increase in peak $\dot{V}O_{2}$ , with area under the ROC curve varying from 0.56 to 0.63, and the best discriminative value for peak $\dot{V}O_{2}$ ml⋅kg^−1^⋅min^−1^ ranging between 17 and 18 ml⋅kg^−1^⋅min^−1^ for these specific complications. We also calculated the ability of peak $\dot{V}O_{2}$ ml⋅kg^−1^⋅min^−1^ to predict post-operative complications according to baseline FEV_1_ (≥ 80% predicted and < 80% predicted). We found that the discriminative ability of peak $\dot{V}O_{2}$ ml⋅kg^−1^⋅min^−1^ was similar between the two FEV_1_ categories with ORs of experiencing post-operative complications of 0.89 (0.81–0.97) and 0.93 (0.86–1.00) per unit of increase in peak $\dot{V}O_{2}$ in the former and latter group, respectively. The corresponding area under the ROC curve was 0.64 and 0.59.

## Discussion

Based on the consistent observation that peak $\dot{V}O_{2}$ during CPET is inversely related to the risk of mortality and morbidity following lung resection (Benzo et al., 2007), the use of CPET has been endorsed by European (Brunelli et al., 2009b) and American (Brunelli et al., 2013) guidelines for risk stratification in lung cancer resection candidates. Data supporting these recommandations were obtained in patients whose lung resection was peformed by thoracotomy (Bolliger et al., 1995; Brutsche et al., 2000; Win et al., 2005; Brunelli et al., 2009a). The present study explored whether these early findings also apply to minimally invasive lung resection performed by VATS. Despite the observation that lower peak $\dot{V}O_{2}$ was associated with a higher likelihood of cardiopulmonary complications after VATS lung cancer resection, the ability of peak $\dot{V}O_{2}$ to predict post-operative cardiopulmonary complications in individual patients was limited. This was true for a composite of cardiopulmonary morbidity and mortality as well as for respiratory and cardiac complications, separately and for the more severe complications (grade III or more).

The past 20 years has witnessed a consistent trend in better post-operative outcome after lung cancer resection, with 30-day post-operative mortality decreasing from 3–5% to 1% (Morgant et al., 2015; Shewale et al., 2020). Improved outcome following lung resection is related to the use of minimally-invasive operative techniques that are associated with less complications than thoracotomy (Boffa et al., 2014; Laursen et al., 2016), but also to improved patient selection, anesthetic techniques, and general postoperative medical management (Morgant et al., 2015; Shewale et al., 2020). Although it is difficult to compare post-operative outcome across studies due to differences in patient populations and in how post-operative complications are documented and defined, our results are consistent with the published experience with VATS lung cancer resection (Boffa et al., 2014; Begum et al., 2016; Shewale et al., 2020).

Considering the lower complication rates that is now observed after lung resection, a lower discriminative ability could be expected from preoperative evaluating tests. This has been suggested by Berry *et al* who reported that pulmonary function tests lose their predictive ability for pulmonary complications in patients who underwent thoracoscopy for lung resection (Berry et al., 2010). Nonetheless, our results show that despite relatively low mortality and morbidity rates of 0.5 and 15.5%, respectively, peak $\dot{V}O_{2}$ was still inversely related to the occurrence of postoperative outcomes. Indeed, peak $\dot{V}O_{2}$ emerged as an independent predictor of mortality and morbidity in the multivariable analysis and there was a significant trend in lower complication rates with progressively higher peak $\dot{V}O_{2}$ as indicated by a prevalence rate of 24.8, 16.3, and 10.4% for peak $\dot{V}O_{2}$ of <15, 15 to <20, and ≥20 ml⋅kg^−1^⋅min^−1^, respectively. Despite this, the ability of peak $\dot{V}O_{2}$ to discriminate between patients who experienced or not an adverse outcome was limited. The area under the ROC curve for peak $\dot{V}O_{2}$ was only 0.63 (95% CI: 0.57–0.69), below the 0.7 threshold that would be considered acceptable for a diagnostic tool (Mandrekar, 2010). Thus, according to the area under the ROC curve, the odds of misclassifying a patient for the occurrence of post-operative cardiopulmonary complications would be 37% ([1—area under the curve]*100). Further, the proportion of patients who experienced a post-operative complication and who had a peak $\dot{V}O_{2}$ < 17 ml⋅kg^−1^⋅min^−1^ (true positive rate) was 56.5% and the proportion of patients who did not experience a post-operative complication and who had a peak $\dot{V}O_{2}$ < 17 ml⋅kg^−1^⋅min^−1^ (false positive rate) was 34.3%. The role of CPET in risk stratifying for VATS was also questioned by Begum *et al* who reported similar postoperative morbidity and mortality after VATS lobectomy for patients with high (≥ 15 ml⋅kg^−1^⋅min^−1^) versus low (< 15 ml⋅kg^−1^⋅min^−1^) peak $\dot{V}O_{2}$ (Begum et al., 2016).

Besides peak $\dot{V}O_{2}$ , numerous CPET findings were associated with complication rates in the present cohort. Lower peak work rate, peak $\dot{V}E$ and peak heart rate, and higher peak $\dot{V}E$ / $\dot{V}CO_{2}$ were all associated with higher complication rate, but only the latter remained significantly associated with post-operative outcome in the multivariable analysis. This parameter had previously been described as a good predictor of postoperative complications in thoracotomy patients (Torchio et al., 2010; Brunelli et al., 2012). Although this finding makes sense from a physiological perspective, the small difference noted between patients with and those without complications makes peak $\dot{V}E$ / $\dot{V}CO_{2}$ difficult to apply to predict of postoperative outcomes.

Strengths of the current study include its large sample size of patients who underwent VATS lung resection and in whom comprehensive CPET data were available. All exercise tests were carried out in the same clinical exercise laboratory and their validity was confirmed by three investigators. We acknowledge that our study has several limitations including its retrospective nature. To minimize the possibility of under reporting complications, we relied on a research database where clinical data is entered prospectively, and this was complemented by the review of medical charts, when necessary. Because only four patients with peak $\dot{V}O_{2}$ below 10 ml⋅kg^−1^⋅min^−1^ were included in this cohort, our results do not necessarily apply to patients with very poor exercise tolerance. The retrospective nature of our study did not allow to retrieve nadir $\dot{V}E$ / $\dot{V}CO_{2}$ values which could have been more informative to predict the occurrence of post-operative complications that $\dot{V}E$ / $\dot{V}CO_{2}$ at peak exercise. By design, only patients who had surgery were considered for this study and we cannot exclude that CPET results may have led to recommending medical treatment in some patients with poor CPET performance. Nevertheless, 105 patients with a peak $\dot{V}O_{2}$ < 15 ml⋅kg^−1^⋅min^−1^ that would classify them as intermediate risk according to current guidelines (Brunelli et al., 2009b; Brunelli et al., 2013) were included, supporting the external validity of the results. Another limitation is the study sample which represents only 27% of the total VATS lung resection population in our institution. The present study represents a real-life situation where the decision to order a CPET pre-operatively was left at the discretion of the surgeons according to their clinical judgment regarding risk categorization, without the use of standardized protocol which would have been preferable from a methodological standpoint considering that CPET could be more discriminative in certain subsets of patients that remain to be identified. It is nevertheless informative to note that in the present study, peak $\dot{V}O_{2}$ performed similarly in patients with FEV_1_ < and ≥ 80% predicted. Lastly, the generalizability of our data is uncertain given that we are reporting a single centre experience.

## Conclusion

Although peak $\dot{V}O_{2}$ was associated with postoperative outcomes in this cohort of patients who underwent lung cancer VATS lung resection, its ability to discriminate for the occurrence of post-operative cardiopulmonary complications was limited. Based on these results, patients should not be denied lung resection on the basis of peak $\dot{V}O_{2}$ when minimally invasive resection is feasible. Prospective studies incorporating CPET results in the preoperative algorithm are needed to better define the place of peak $\dot{V}O_{2}$ in the evaluation of patients undergoing VATS resection for lung cancer.

## Data Availability

The raw data supporting the conclusions of this article will be made available anonymously on request without undue reservation.
